# Orbital complications of paranasal sinusitis in Taiwan, 1988 through 2015: Acute ophthalmological manifestations, diagnosis, and management

**DOI:** 10.1371/journal.pone.0184477

**Published:** 2017-10-03

**Authors:** Yi-Sheng Chang, Po-Lin Chen, Jia-Horung Hung, Hsiao-Yen Chen, Chun-Chieh Lai, Chun-Yen Ou, Chia-Ming Chang, Chien-Kuo Wang, Hon-Chun Cheng, Sung-Huei Tseng

**Affiliations:** 1 Department of Ophthalmology, College of Medicine, National Cheng Kung University, Tainan, Taiwan; 2 Department of Ophthalmology, National Cheng Kung University Hospital, College of Medicine, National Cheng Kung University, Tainan, Taiwan; 3 Department of Medicine, College of Medicine, Kaohsiung Medical University, Kaohsiung, Taiwan; 4 Institute of Clinical Medicine, College of Medicine, National Cheng Kung University, Tainan, Taiwan; 5 Department of Otorhinolaryngology, National Cheng Kung University Hospital, College of Medicine, National Cheng Kung University, Tainan, Taiwan; 6 Division of Geriatrics and Gerontology, Department of Internal Medicine, National Cheng Kung University Hospital, College of Medicine, National Cheng Kung University, Tainan, Taiwan; 7 Division of Infectious Diseases, Department of Internal Medicine, National Cheng Kung University Hospital, College of Medicine, National Cheng Kung University, Tainan, Taiwan; 8 Center of Infection Control, National Cheng Kung University Hospital, College of Medicine, National Cheng Kung University, Tainan, Taiwan; 9 Department of Radiology, National Cheng Kung University Hospital, College of Medicine, National Cheng Kung University, Tainan, Taiwan; Aligarh Muslim University, INDIA

## Abstract

**Purpose:**

Paranasal sinusitis is widespread and can lead to orbital complications, blindness, and death. However, the correlation between ophthalmological findings and disease staging remains unclear. This study aimed to investigate the staging, acute ophthalmological manifestations, diagnosis, management, and outcomes of orbital complications of paranasal sinusitis during a 27-year period.

**Methods:**

We retrospectively reviewed the medical records of all patients with orbital complications of paranasal sinusitis hospitalized at the National Cheng Kung University Hospital, a medical center in Taiwan during 1988–2015. Sex, age, symptoms, history, ophthalmological findings, laboratory and imaging findings, treatments, and outcomes were analyzed by staging.

**Results:**

Eighty-three patients aged 9 days to 80 years had stage I (preseptal cellulitis, n = 39 patients), II (postseptal orbital cellulitis, n = 8), III (subperiosteal abscess, n = 16), IV (orbital abscess, n = 8), or V (intracranial involvement, n = 12) complications. Peak incidences occurred in patients aged 0–19 and 60–69 years. Chronic sinusitis and diabetes mellitus were common preexisting diseases. Extraocular movement limitation and proptosis predicted postseptal (stage II or more) involvement. The likelihood of elevated intraocular pressure increased with stage. Reduced visual acuity and presence of relative afferent pupillary defect indicated consideration of magnetic resonance imaging to investigate possible intracranial extension. Ipsilateral maxillary (81.7%) and ethmoidal (75.6%) sinuses were the most common sources of infection, and the most frequently implicated pathogens were coagulase-negative *Staphylococcus* spp. (25.3%) and *Staphylococcus aureus* (20.5%). All patients received intravenous antimicrobial therapy (multi-drug therapy in 88.0%), and 55.4% underwent surgery, most commonly endoscopic sinus surgery. One (1.2%) diabetic man with stage V complications died of fungal sinusitis with intracranial invasion. Five (6.0%) patients, all stage V, lost vision despite intensive treatment. The average length of hospital stay was 13.8 days (range 2–72 days), and significantly longer stays were associated with stages II–V as compared to stage I.

**Conclusions:**

Orbital infection originating from paranasal sinusitis can cause vision loss and death due to intracranial extension. Acute ophthalmological findings predict staging and prognosis. Cooperative consultation between ophthalmologists, otorhinolaryngologists, and neurologists is essential. Urgent diagnostic studies and aggressive antimicrobial therapy are indicated, and surgery should be considered.

## Introduction

Paranasal sinusitis is widespread, with an annual prevalence of 13.0–16.0% in the United States [1], 10.9% (range 6.9–27.1%) in Europe [2], and 8.0% (range 4.2–10.2%) in China [3]. Its most common complication, orbital infection, occurs when pathogens pass from an infected maxillary, ethmoidal, frontal, or sphenoidal sinus into the orbit, either directly though neurovascular foramina or a congenital or acquired bony dehiscence, or indirectly through valveless veins of the sinuses and orbit.

The process involves edema of the sinus mucosa, which narrows the ostia and impairs sinus drainage. Bacterial or fungal microflorae in the sinuses proliferate and invade the edematous mucosa, resulting in suppuration. It is augmented by reduced oxygen tension within an obstructed sinus cavity. Then these organisms enter the orbit, leading to preseptal or orbital inflammation. Moreover, subperiosteal or orbital abscesses may occur. The resulting elevation of intraorbital pressure results in periorbital swelling, proptosis, ophthalmoplegia, chemosis, and optic nerve compression. Furthermore, some may extend to the brain leading to inflammation, abscess formation, or cavernous sinus thrombosis.

Although bacteria are more commonly seen in acute sinusitis. fungal infections usually occur in immunocompromised individuals. While antimicrobial therapy has reduced the risks of permanent sequelae, orbital involvement can still lead to blindness, or where intracranial extension occurs, death. We aimed to investigate the clinical features of orbital complications of sinusitis in a Taiwanese population, and observed that certain ophthalmological manifestations and outcomes were significantly associated with disease stage.

## Patients and methods

### Patients

Medical records of all patients diagnosed with orbital complications of sinusitis hospitalized at the National Cheng Kung University Hospital (NCKUH) in Taiwan between January 1988 and December 2015 were reviewed. The inclusion criteria of our study were inpatients of all age groups presenting with preseptal/orbital inflammation secondary to acute (<1 month), subacute (1–3 months) or chronic sinusitis (>3 month) of bacterial, fungal or unidentified pathogens. The diagnosis was based on clinical symptoms and signs as well as laboratory tests and radiological investigations. Sinusitis was confirmed by the presence of sinus opacification or air-fluid levels on computerized tomography (CT) or X-ray when CT was not available. The exclusion criteria were hematological bone marrow disorders and sinus/orbital cancers. The data recorded included sex, age, symptoms, medical history, ophthalmic examination findings, systemic and laboratory findings, imaging results and the sinuses involved, culture results, treatments, hospital stay durations, and outcomes. This retrospective study was approved by the Institutional Review Board of NCKUH (A-ER-102-336), which waived the need for informed consent because patient anonymity was inherent in the data source.

### Staging

In this study, orbital complications of sinusitis were classified via a modified form of the classification system reported by Chandler et al [4]. Focal thickening and infiltration of the eyelid anterior to the orbital septum were classified as stage I, preseptal cellulitis. Edema and inflammation of the orbital contents without evidence of abscess formation were classified as postseptal stage II, orbital cellulitis. Abscess formation between the orbital wall and the periorbita was classified as stage III, subperiosteal abscess. Abscess formation with pus or debris within the orbital content was classified as stage IV, orbital abscess. Additional intracranial extension, including cavernous sinus thrombosis, meningitis, cerebritis, or epidural/subdural/intracerebral abscess or empyema, was classified as stage V [5–7].

### Statistics

Categorical variables were analyzed using the Chi-square test or Fisher’s exact test, and continuous variables were analyzed using Student’s *t*-test, via SPSS software (version 20, IBM, Armonk, New York, USA). A *P* value < 0.05 was considered statistically significant.

## Results

**Fig 1:**
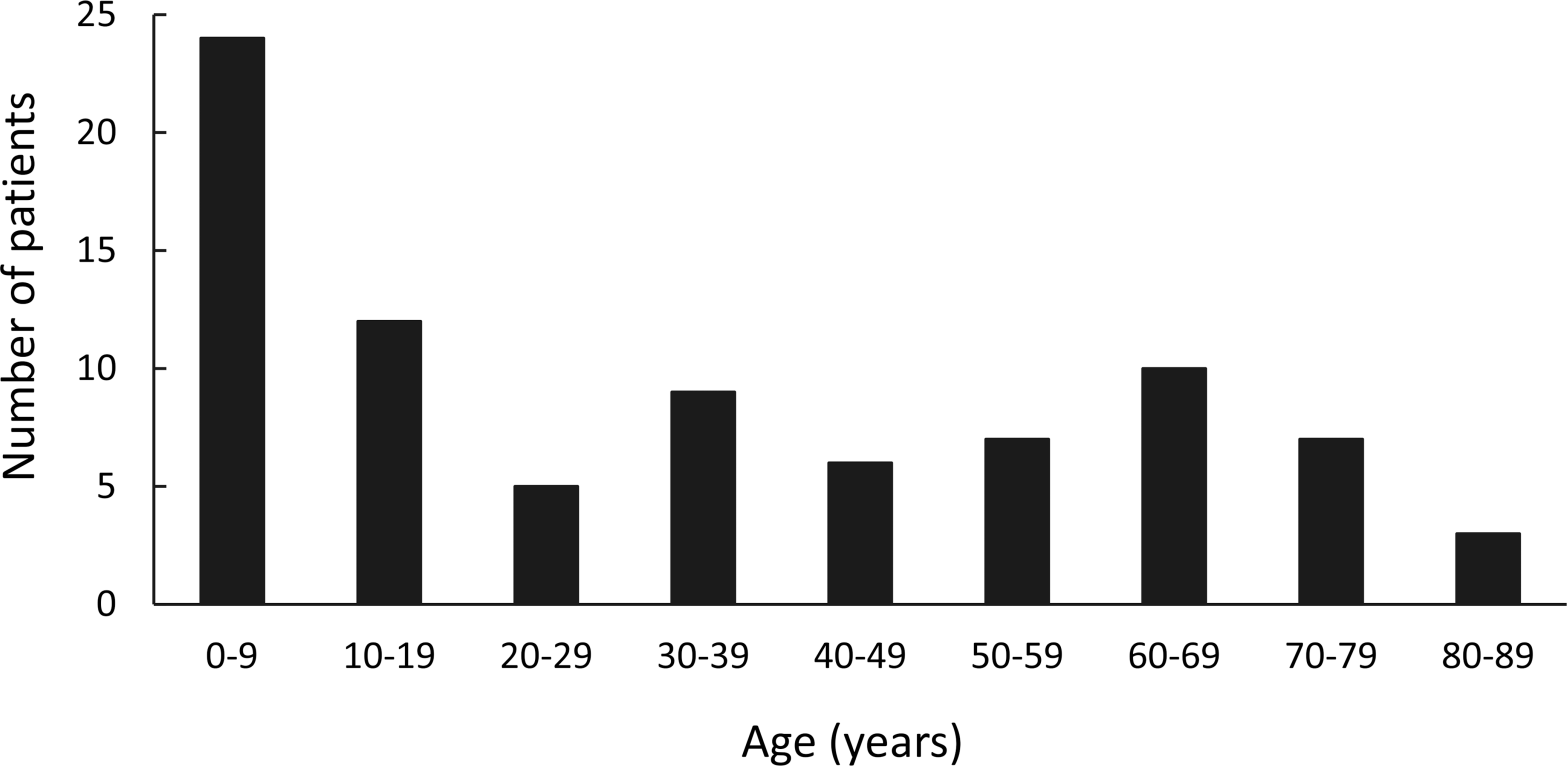
Age distribution of 83 patients with orbital complications of sinusitis. Age peaked at 0–9 years, followed by 10–19 years. An additional peak occurred at 60–69 years.

Eighty-three patients (35 male, 48 female) aged 9 days to 80 years (mean 33.7, SD 26.4 years) were identified. Younger patients were most commonly afflicted, with 24 (28.9% of the total) aged 0–9 years, followed by 12 (14.5%) patients aged 10–19 years, though notably there was an additional peak at 60–69 years (10, 12.0%) (**Fig 1**).

### Clinical findings

**Table 1**presents clinical findings classified by stage of sinusitis-related orbital complications. Thirty-nine (47.0%) of the 83 patients had preseptal disease (stage I), 32 (38.6%) had postseptal disease (stages II–IV), and 12 (14.5%) had intracranial involvement (stage V). Seventy-one (85.5%) presented symptoms typical of upper respiratory tract infections (*e*.*g*., nasal obstruction, mucopurulent drainage, headache, fever, fatigue, or cough) before admission, and 5 (6.2%) had experienced traumatic blowout fracture in the last 3 weeks. Thirty (36.1%) had a history of chronic sinusitis (45.8% in adults versus 22.9% in children; *P* = 0.03), 20 (24.1%) had diabetes mellitus, 3 (3.6%) adults had chronic illnesses (including end stage renal disease, hepatitis C-related liver cirrhosis, or poliomyelitis-related paraplegia), 2 (2.4%) children had asthma, and 1 (1.2%) child had an atrial septal defect. Diabetes mellitus was associated with disease stage, with 10.3% in stage I, 28.1% in stages II–IV, and 58.3% in stage V (*P* = 0.006, stages II–V versus stage I), but chronic sinusitis was borderline with regard to significance (*P* = 0.06).

**Table 1:** Demographic characteristics and clinical findings in 83 patients by stage of orbital complications of sinusitis.

	Stage, Number (%)						
	Preseptal	Postseptal			Intracranial		
	I	II	III	IV	V	Total	
Characteristics / finding	39 (47.0)	8 (9.6)	16 (19.3)	8 (9.6)	12 (14.5)^b^	83 (100)	*P* value^a^
**Sex**							0.81
Male	17 (43.6)	3 (37.5)	6 (37.5)	2 (25.0)	7 (58.3)	35 (42.2)	
Female	22 (56.4)	5 (62.5)	10 (62.5)	6 (75.0)	5 (41.7)	48 (57.8)	
**Age group**							0.49
Adult	21 (53.8)	7 (87.5)	8 (50.0)	4 (50.0)	8 (66.7)	48 (57.8)	
Child (≤ 19 years)	18 (46.2)	1 (12.5)	8 (50.0)	4 (50.0)	4 (33.3)	35 (42.2)	
**Age (years)**							
Mean ± SD	30.0 ± 26.6	54.7 ± 19.9	31.3 ± 25.1	25.6 ± 28.5	40.3 ± 28.0	33.7 ± 26.4	0.55
Range	1–80	0–77	2–73	2–69	0–73	0–80	
**URI-like symptoms**	32 (82.1)	8 (100.0)	15 (93.8)	6 (75.0)	10 (83.3)	71 (85.5)	0.39
**History**							
Chronic sinusitis	10 (25.6)	4 (50.0)	7 (43.8)	4 (50.0)	5 (41.7)	30 (36.1)	0.06
Diabetes mellitus	4 (10.3)	5 (62.5)	3 (18.8)	1 (12.5)	7 (58.3)	20 (24.1)	0.006
**Ophthalmological finding**							
EOM limitation	6 (15.4)	6 (75.0)	13 (81.3)	6 (75.0)	9 (75.0)	40 (48.2)	< 0.001
Proptosis	4 (10.3)	5 (62.5)	10 (62.5)	6 (75.0)	7 (58.3)	32 (38.6)	< 0.001
IOP > 23 mmHg	4 (10.3)	2 (25.0)	6 (37.5)	3 (37.5)	5 (41.7)	20 (24.1)	0.006
VA change^c^	3 (7.7)	1 (12.5)	3 (18.8)	4 (50.0)	10 (83.3)	21 (25.3)	0.001
RAPD present	0	0	1 (6.3)	1 (12.5)	6 (50.0)	8 (9.6)	0.005
**Systemic finding**							
Fever (> 37.5°C)	19 (48.7)	3 (37.5)	9 (56.3)	6 (75.0)	8 (66.6)	45 (54.2)	0.34
Leukocytosis (> 10,000/mm^3^)	27 (69.2)	4 (50.0)	10 (62.5)	6 (75.0)	10 (83.3)	57 (68.7)	0.91
CRP > 10.0 mg/L	31 (79.5)	8 (100.0)	12 (75.0)	7 (87.5)	10 (83.3)	68 (81.9)	0.59
**Sinusitis**^d^							
Maxillary							
Ipsilateral	31 (79.5)	5 (62.5)	12 (75.0)	8 (100.0)	12 (100.0)	68 (81.9)	0.59
Contralateral	14 (35.9)	1 (12.5)	3 (18.8)	3 (37.5)	5 (41.7)	26 (31.3)	0.40
Ethmoidal							
Ipsilateral	24 (61.5)	7 (87.5)	14 (87.5)	8 (100.0)	10 (83.3)	63 (75.9)	0.004
Contralateral	8 (20.5)	1 (12.5)	3 (18.8)	3 (37.5)	7 (58.3)	22 (26.5)	0.24
Frontal							
Ipsilateral	15 (38.5)	0	11 (68.8)	3 (37.5)	5 (41.7)	34 (41.0)	0.66
Contralateral	6 (15.4)	0	3 (18.8)	1 (12.5)	1 (8.3)	11 (13.3)	0.59
Sphenoidal							
Ipsilateral	4 (10.3)	0	0	3 (37.5)	4 (33.3)	11 (13.3)	0.45
Contralateral	2 (5.1)	0	0	2 (25.0)	3 (25.0)	7 (8.4)	0.31

**Abbreviations:** CRP, C-reactive protein; EOM, extraocular movement; IOP, intraocular pressure; RAPD, relative afferent pupillary defect; SD, standard deviation; URI, upper respiratory tract infection; VA, visual acuity.

^a^ Comparison between stage I and advanced stages (II–V).

^b^ Stage V: cavernous sinus thrombosis in 5 patients, frontal lobe abscesses in 4, and meningitis in 3.

^c^ VA change: reduction in visual acuity of ≥ 2 lines compared with fellow eye or baseline.

^d^ Imaging evidence of sinusitis, by side of ocular complications

**Fig 2:**
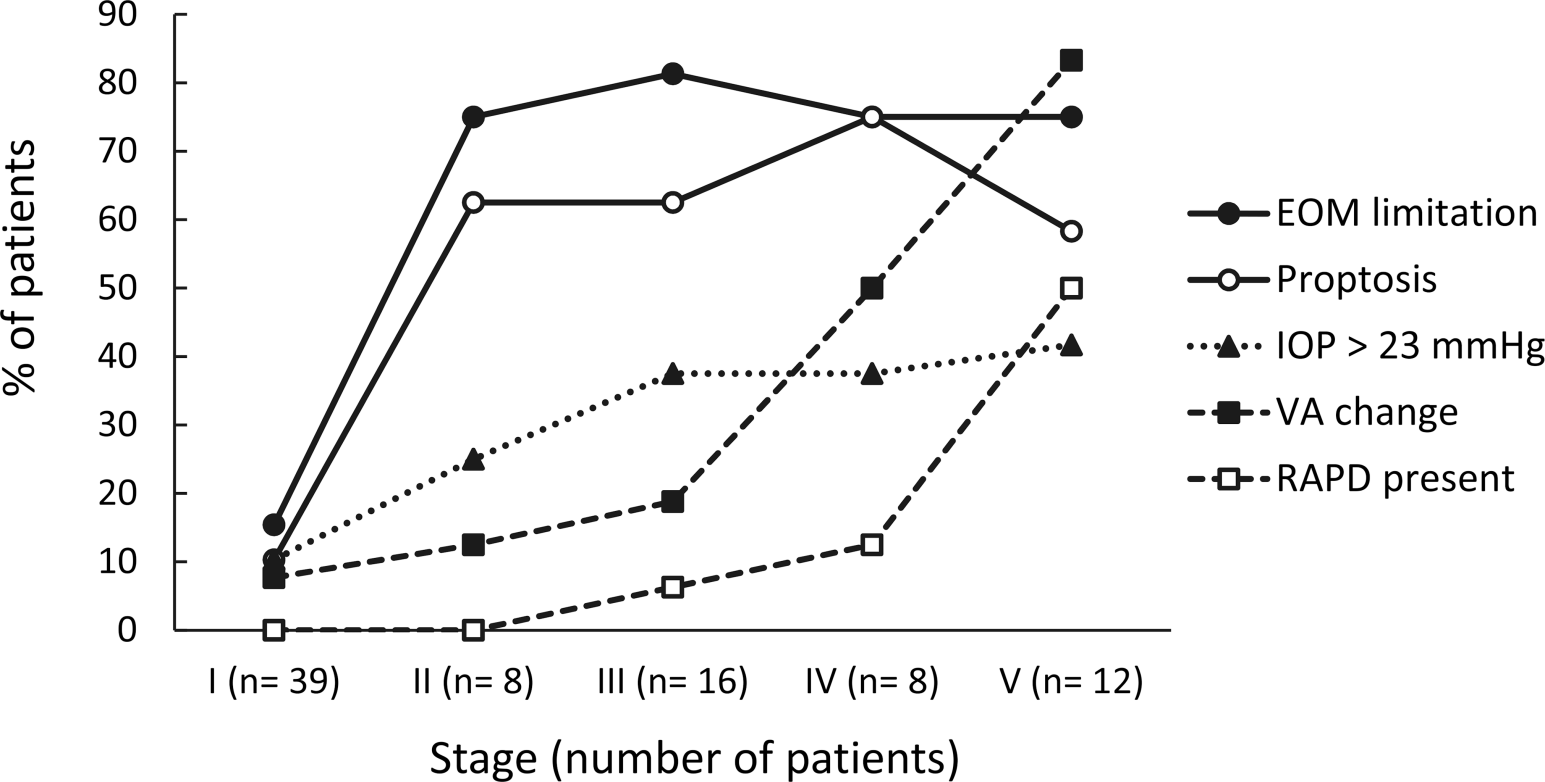
Percentages of acute ophthalmological findings in 83 patients by stage of orbital complications of sinusitis. The increasing incidences of acute ophthalmological findings by stage were categorized into three patterns. “EOM limitation” and “proptosis” substantially increased from stage II, and thereafter were sustained at high percentages during stages III–V. “IOP > 23 mmHg” increased steadily by stage. The incidences of “VA change” and “RAPD present” substantially increased at stages IV and V respectively. **Abbreviations:** EOM, extraocular movement; IOP, intraocular pressure; RAPD, relative afferent pupillary defect; VA, visual acuity.

All patients had periorbital swelling. As shown in **Table 1**and **Fig 2**, clinical findings that predicted more severe disease (stages II–V versus stage I) were limitation of extraocular movement (EOM), proptosis, elevated intraocular pressure (IOP), reduced visual acuity, and relative afferent pupillary defect (RAPD) (all *P*s < 0.01). The changes in these ophthalmological parameters with stage were observed in three patterns. First, the incidences of EOM limitation and proptosis substantially increased from stage II, and thereafter were sustained at high percentages in stages III–V. Second, the incidence of IOP elevation increased steadily with stage. Third, the incidences of reduced visual acuity and RAPD substantially increased at stages IV and V respectively.

Fever was present in 24 (68.6%) of 35 children (≤ 19 years) versus 21 (43.8%) of 48 adults (*P* < 0.001), and was observed in only 7 (35.0%) of those aged 60 years or older (*P* = 0.006; children versus those aged 60 years or older). Leukocytosis and elevated C-reactive protein were seen in 68.7% and 81.9% of subjects respectively, but were not associated with disease severity.

**Fig 3:**
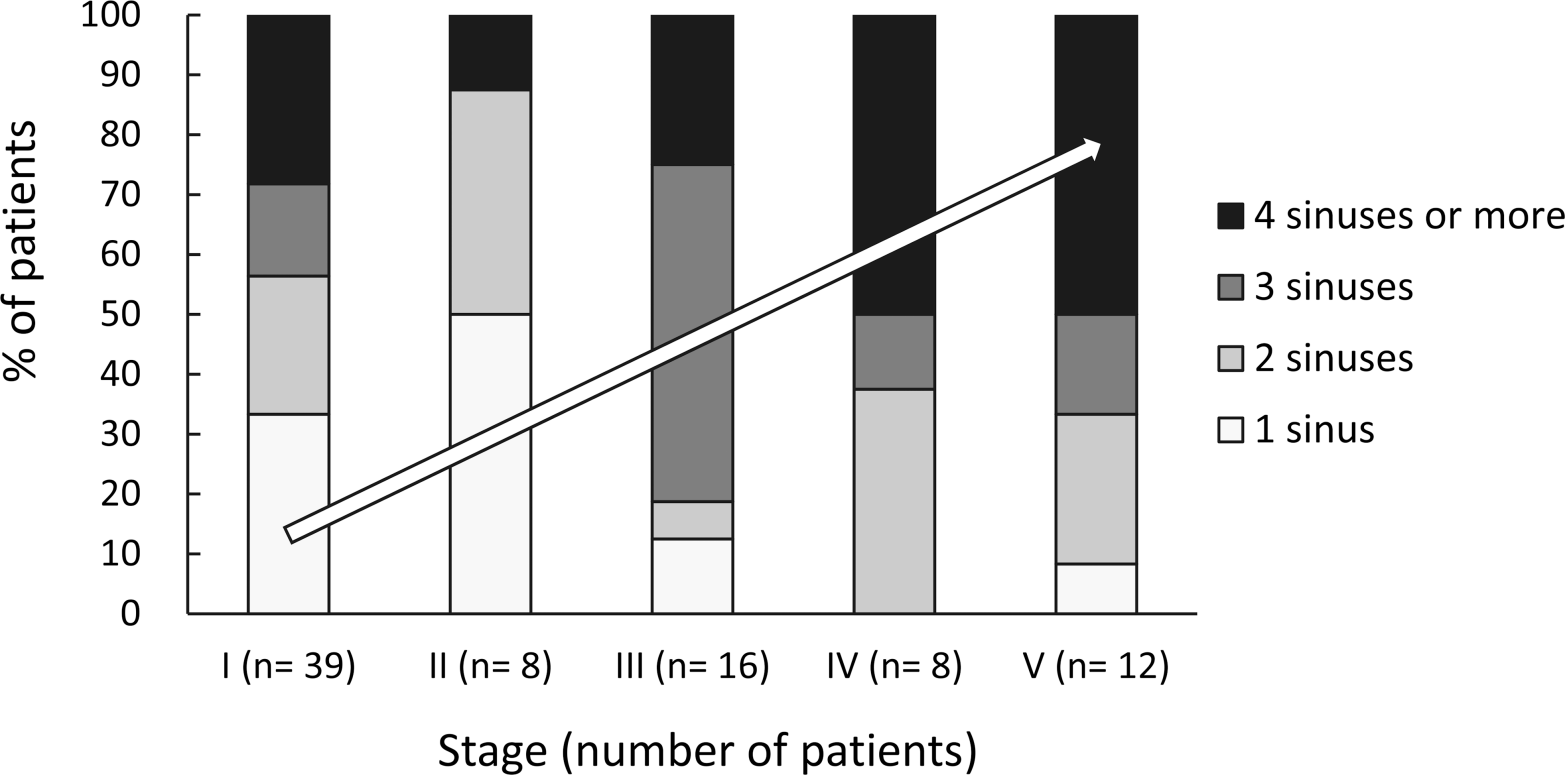
Percentages of sinuses involved in 83 patients by stage of orbital complications of sinusitis. There was a trend of a positive association between stage and the number of sinuses involved. The most frequently involved numbers of sinuses were 1 for stages I and II, 3 for stage III, and 4 or more for stages IV and V.

**Fig 4:**
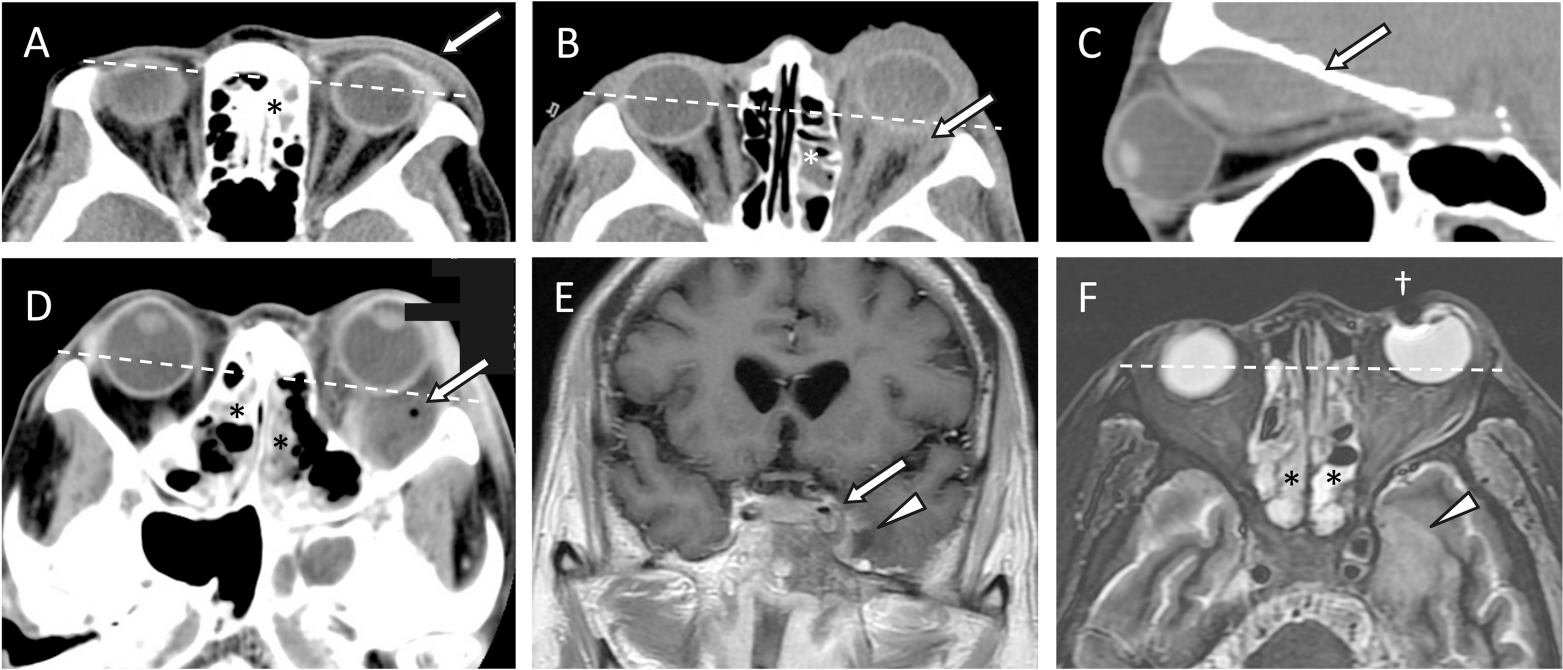
Characteristic findings of each stage of sinusitis-related orbital complications determined via computed tomography (CT) or magnetic resonance imaging (MRI). **(A)** Stage I, preseptal cellulitis (arrow) on an axial CT image. **(B)** Stage II, orbital cellulitis (arrow) on an axial CT image. **(C)** Stage III, subperiosteal abscess (arrow) on a sagittal CT image. **(D)** Stage IV, orbital abscess (arrow) with a tiny air bubble on an axial CT image. **(E and F)** Stage V, cavernous sinus thrombosis (arrow) on a coronal contrast-enhanced T1-weighted MRI, and focal cerebritis (arrowheads) on a coronal contrast-enhanced T1-weighted MRI and an axial T2-weighted MRI. * Ethmoidal sinusitis. † Artifact due to metal material. Dashed lines: Proptosis in one eye compared with the fellow eye.

CT of orbits and sinuses was conducted in 71 (85.5%) patients, and sinusitis and preseptal/orbital inflammation was evident in all these cases. The remaining 12 patients (10 children and 2 adults, all stage I), underwent only Waters’ view X-ray. Magnetic resonance imaging (MRI) was performed in 11 (13.3%) patients, all stage V, due to suspected intracranial extension. As shown in **Table 1**, the ipsilateral maxillary (68 patients, 81.9%) and ipsilateral ethmoidal (63 patients, 75.9%) sinuses were the most commonly involved. Twenty (24.4%) patients had only 1 sinus involved, and the rest had 2–6 sinuses involved. The involvement of more sinuses was associated with more severe orbital disease stages (**Fig 3**). **Fig 4**shows the characteristic imaging findings for each stage of orbital complications.

Cultures from the sinuses, orbit, or brain in 48 (57.8%) patients yielded the growth of a single pathogen (25 cases; 30.4%) or polymicrobial pathogens (23 cases; 27.7%) (**Table 2**). The most frequently implicated pathogens were coagulase-negative *Staphylococcus* spp. (25.3%) and *Staphylococcus aureus* (20.5%). Blood cultures were positive in 6 (7.3%) patients, including three aged 9 days, 23 days, and 2 years.

**Table 2:** Pathogens isolated from the sinuses, orbit, or brain in 48 of 83 patients with orbital complications of sinusitis.

Pathogen	Number (% of 83) patients^a^
**Aerobes**	
Coagulase-negative *Staphylococcus* spp.	21 (25.3)
*Staphylococcus aureus*	17 (20.5)
*Pseudomonas aeruginosa*	6 (7.2)
*Viridans Streptococcus* group	6 (7.2)
*Streptococcus pneumoniae*	5 (6.0)
*Klebsiella* spp.	4 (4.8)
*Enterococcus* spp.	4 (4.8)
*Bacillus* spp.	4 (4.8)
*Enterobacter* spp.	3 (3.6)
*Neisseria* spp.	2 (2.4)
*Escherichia coli*	2 (2.4)
*Acinetobacter baumanii*	1 (1.2)
*Citrobacter diversus*	1 (1.2)
*Flavobacterium* spp.	1 (1.2)
*Morganella moganii*	1 (1.2)
**Anaerobes**	
*Prevotella* spp.	3 (3.6)
*Peptostreptococcus micros*	2 (2.4)
*Propionibacterium* spp.	2 (2.4)
*Haemophilus influenzae*	2 (2.4)
*Fusobacterium* spp.	1 (1.2)
*Gemella* spp.	1 (1.2)
**Fungi**	
Yeast	5 (6.0)
*Aspergillus* spp.	3 (3.6)

^a^ A single pathogen in 25 cases, and polymicrobial infection in 23 cases.

### Treatments

All patients received intravenous antimicrobials, 9 (10.8%) with amoxicillin/clavulanate alone, 1 (1.2%) with ampicillin/sulbactam alone, and the other 73 (88.0%) with multiple antimicrobials. The most frequently administered antimicrobials were amoxicillin/clavulanate used in 45 (54.2%) patients, gentamicin in 25 (30.1%), and ampicillin/sulbactam in 21 (25.3%).

Surgery was performed in 46 (55.4%) patients (**Table 3**), including endoscopic sinus surgery in 38 (45.8%), orbitotomy in 3 (3.6%), frontoethmoidectomy in 2 (2.4%), craniotomy in 2 (2.4%), and Caldwell-Luc antrostomy in 1 (1.2%). Surgery was performed in 16 (34.0%) of the patients with stage I–II complications versus 30 (83.3%) of those with stage III–V complications (*P* < 0.001), and in 19 (54.3%) children versus 27 (56.3%) adults (*P* = 0.86). The mean (± SD) time from admission to surgery was 2.4 (± 5.6) days (range 0–21 days).

**Table 3:** Treatments and outcomes in 83 patients by stage of orbital complications of sinusitis.

	Stage, Number (%)						
	Preseptal	Postseptal			Intracranial		
	I	II	III	IV	V	Total	
Treatment / outcome	39 (47.0)	8 (9.6)	16 (19.3)	8 (9.6)	12 (14.5)	83 (100)	*P* value^a^
**Treatment**							
Number (%) undergoing surgery	13 (33.3)	3 (37.5)	13 (81.3)	6 (75.0)	11 (91.7)	46 (55.4)	< 0.001
Days from admission to surgery							
Mean ± SD	1.3 ± 1.7	2.0 ± 1.2	1.9 ± 1.7	2.0 ± 1.6	7.1 ± 7.0	2.4 ± 5.6	0.11
Range	0–6	0–4	0–2	0–5	1–21	0–21	
Days of hospitalization							
Mean ± SD	7.0 ± 3.8	22.1 ± 18.6	11.2 ± 4.3	19.1 ± 18.9	30.2 ± 22.5	13.8 ± 13.9	< 0.001
Range	2–20	5–49	5–22	5–62	6–72	2–72	
**Outcome**							
Death	0	0	1 (6.3)^b^	0	1 (8.3)^c^	2 (2.4)	0.18
Blindness	0	0	0	0	5 (41.7)^d^	5 (6.0)	0.03

**Abbreviations:** CRP, C-reactive protein; EOM, extraocular movement; IOP, intraocular pressure; RAPD, relative afferent pupillary defect; SD, standard deviation; URI, upper respiratory tract infection; VA, visual acuity.

^a^ Comparison between stage I and advanced stages (II–V).

^b^ Cause of death was recurrent episodes of pneumonia and respiratory failure during admission, due to preexisting chronic immobility resulting from long-standing paralysis, and was considered unrelated to orbital complications of sinusitis.

^c^ Cause of death was intracranial invasion to the left cavernous sinus and left medial temporal lobe, resulting from invasive fungal sinusitis and orbital complications.

^d^ No light perception in 2 patients and counting fingers in 3 patients.

### Outcomes

The mean (± SD) hospital stay was 13.8 (± 13.9) days (range 2–72 days) (**Table 3**), and was significantly longer in cases of more severe disease (*P* < 0.001; stages II–IV versus stage I). Recovery was graded as complete in 76 (92.7%) patients, including 3 children too young to measure visual acuity whose periorbital inflammation resolved completely. A 60-year-old diabetic man with stage V disease presented with fungal sinusitis and orbital complications, and died of intracranial invasion to the left cavernous sinus and left medial temporal lobe within 15 days of hospitalization. One stage III patient with preexisting paraplegia due to poliomyelitis died of recurrent episodes of pneumonia and respiratory failure considered unrelated to ophthalmic complications of sinusitis. Five patients (all stage V) lost vision permanently in the affected eye, despite intensive treatment.

## Discussion

To our knowledge, this is the first study analyzing disease stage and risk factors, manifestations, diagnosis, treatments, and outcomes associated with orbital complications of sinusitis conducted in an Asian population. We detected incidence peaks in patients aged 0–19 and 60–69 years. Chronic sinusitis and diabetes mellitus were common preexisting diseases. EOM limitation and proptosis predicted postseptal involvement, and reduced visual acuity and the presence of RAPD indicated that intracranial extension was likely. Maxillary and ethmoidal sinuses were common sources of infection, and the number of sinuses involved tended to increase with stage. Staphylococci were common pathogens. More than a half of patients underwent surgery. Among the 12 patients with stage V complications, 1 died and 5 lost vision in the affected eyes.

### Age at diagnosis and preexisting diseases

Acute sinusitis is the most common cause of orbital infections in children [8], and we found a peak incidence of orbital complications in this age group. This is concordant with results reported by Ferguson and McNab [9], and may be attributable to incomplete paranasal sinus development and thinner bony barriers in such patients. In contrast, acute exacerbation of chronic sinusitis is often the cause of serious complications in adults, and a history of chronic sinusitis was identified in 45.8% of adults versus 22.9% of children in this study. Predisposing factors of sinusitis include anatomic derangements, impaired sinus drainage, and inhibition of mucociliary transport, which promote bacterial overgrowth [10]. In addition, immunocompromised states (*e*.*g*., diabetes mellitus, chronic renal failure, chronic liver disease, high-dose steroid therapy, or acquired immune deficiency syndrome) reportedly predispose patients to bacterial sinusitis and orbital cellulitis [5]. In the current study, diabetes mellitus was present in 24.1% of patients, liver cirrhosis in 1.2%, and end stage renal disease in 1.2%. We attributed the observed peak in the patients aged 60–69 (and older) to immunosuppression due to aging, diabetes mellitus, or other chronic systemic diseases.

### Ophthalmological but not systemic findings as indicators of disease severity

In addition to the periorbital swelling observed in all patients in this study, acute ophthalmological findings complicated by sinusitis included EOM limitation, proptosis, IOP elevation, reduced visual acuity, and RAPD. More severe orbital sinusitis complications were also significantly correlated with these ophthalmological conditions, which is concordant with previously reported studies [9,11–13]. A notable contribution of this study is that we categorized increases in these ophthalmological manifestations by stage into three patterns in an effort to investigate their predictive value with regard to disease staging and prognosis. Generally, the manifestation of only periorbital swelling predicts stage I, while concurrent EOM limitation or proptosis predicts stage II [8]. Additionally, elevation of IOP renders stage III more likely. Visual impairment predicts stage IV, and the development of RAPD raises a higher possibility of stage V, which warrants further intracranial imaging and neurological assessment, and predicts potential blindness and death.

With regard to systemic manifestations, leukocytosis and elevation of C-reactive protein were common in the current study (68.7% and 81.9% respectively), indicating an infected or inflamed state. However, only 54.2% of patients had fever on presentation, and we surmised that some had likely been treated with antimicrobials and/or antipyretic agents by local physicians prior to presentation, as symptoms typical of upper respiratory tract infections were present in most. Notably, the absence of fever does not exclude severe infection, especially in those with reduced immune function due to aging or chronic disease [14]. Our finding of fever in 65.7% of children compared to 43.8% of adults and 35.0% of patients aged 60 years or older is concordant with the results of a study reported by Ferguson and McNab [9], in which 71% of children versus 32% of adults had temperatures > 37.5°C.

### Diagnostic imaging

CT scanning was performed in 85.5% of our patients, and accurately established the diagnosis of sinusitis and the extent of orbital complications. Some have suggested that CT scanning should be reserved for cases in which a postseptal orbital disease is suspected, after 24 hours of medical treatment without improvement, or when vision cannot be accurately assessed [7]. However, Rubin et al. [15] have reported that two patients with sinusitis-related subperiosteal abscess only presented symptoms of eyelid edema and erythema. This supports our recommendation that CT scanning be performed to exclude or confirm sinusitis, either with or without orbital involvement, especially in patients with a history of sinusitis and no history of eyelid trauma. CT is also useful in the planning of surgery, because it delineates extraocular muscles, the optic nerve, orbital walls, and the bony margins of nearby sinuses [6,8,16]. MRI on the other hand, which we performed in 11 of 12 patients with intracranial extension, better delineates intracranial complications such as cavernous sinus thrombosis, meningitis, cerebritis, and epidural/subdural/intracerebral abscess or empyema [6,16].

Our finding that the ipsilateral maxillary and ethmoidal sinuses were the most common sources of infection causing orbital/intracranial complications is concordant with previous studies [11,13,16]. Infection in the ethmoidal sinus may spread directly into the orbit via the thin bone of the lamina papyracea, or indirectly to the brain via septic thrombophlebitis [6]. Ipsilateral frontal sinusitis occurred in 34 (41.0%) of the patients in the current study, but was not observed in those younger than 8 years; which is logical as the frontal sinus does not develop until after the age of 6 years. Notably however, infection in the frontal sinus spreading to the brain via the thin bone of the anterior cranial fossa, resulting in frontal lobe abscess, is reportedly the most common intracranial complication of sinusitis [16].

### Pathogens

Pathogens isolated from our patients’ sinuses, orbit, or brain were most often coagulase-negative *Staphylococcus* spp. and *Staphylococcus aureus*, which is concordant with previous reports [5,9,13]. Anaerobes were isolated in only 5 (6.0%) of our cases, and were usually associated with polymicrobial infection, as has been described in some previous reports [9,17,18]. Harris [19] reported that no growth or growth of a single aerobic species was likely in cultures from patients aged younger than 9 years, and that polymicrobial or anaerobe growth was more likely with increasing age after 9 years. A notable medical improvement that has occurred over time is that *Haemophilus influenzae* type B, *Streptococcus pneumoniae*, and *Viridans Streptococcus* group previously often caused sinusitis or orbital infection in young children, but they are no longer significant causes of such infections following the introduction of relevant vaccines in the years 1985 and 2000 respectively [7,20]; as confirmed by the results of the current study.

Sinus or orbital infection can lead to bacteremia, which is reportedly more common in newborns and infants than in older subjects due to their immature immune systems [21]. This contention is consistent with our finding that 3 of our 6 patients with positive blood cultures were either newborns or young children. In fact, orbital complications of acute sinusitis are very rare in infants, and to date only 11 cases aged 10–74 days have been reported in the literature in the past 50 years [21]. The current study adds 2 cases to this historically documented cohort: one was aged 23 days and exhibited stage II complications, and the other was aged 9 days and exhibited stage V complications. Both were successfully treated and recovered.

### Treatment

While recommendations of antimicrobial treatment for orbital complications of sinusitis vary, most clinicians suggest multi-drug combinations or a single broad-spectrum antimicrobial, to safe-guard against polymicrobial pathogens including anaerobes [7,9]. Some agree that amoxicillin/clavulanate, which has historically often been used (alone or in combination) and was used in our study is effective against beta-lactamase-producing aerobes and anaerobes, is suitable for use in all age-groups [19].

The goals of surgery for orbital complications of sinusitis are to drain the abscess adequately, release pressure in the orbit, and obtain material for culture.^7^ Endoscopic sinus surgery, introduced in the 1980s [22], was the most frequently used procedure in the current study. It has several advantages over an open procedure, including the negation of an external wound, less postoperative edema, and more rapid recovery [18]. The likelihood of surgery increased with more advanced stages of orbital complications in the current study.

It is generally believed that preseptal cellulitis and orbital cellulitis respond to drug treatment alone, but subperiosteal/orbital abscess or intracranial complications require surgical drainage [7–9,15]. Several reports indicate that drug treatment alone may be effective for ethmoidal sinusitis-related subperiosteal abscess in some children aged younger than 9 years with intact visual function, provided they meet certain additional criteria [19,23,24]. Nevertheless, good clinical judgment should always take precedence and emergency drainage of a subperiosteal abscess may be necessary.

### Outcomes and prognosis

Orbital complications of sinusitis can lead to blindness and death. Causes of vision-loss include: (1) optic neuritis resulting from a reaction to an adjacent or nearby infection, (2) ischemia resulting from thrombophlebitis along the valveless orbital veins, and (3) pressure ischemia, possibly causing central retinal artery occlusion [15–17]. Prompt decompression of the orbit in such cases may preserve the patient’s vision. The rate of blindness in the current study was 6.0%, which is statistically comparable with that of 2.5% reported by Patt and Manning [25]. Notably, blindness was exclusively and commonly (41.7%) observed in conjunction with stage V disease in the current study. Importantly, death resulting from orbital complications of sinusitis is rare if the infection is appropriately treated [21,26]. However, stage V disease can deteriorate rapidly and lead to death, as occurred in the patient in the current study who expired within 15 days of hospitalization.

### Study advantages and limitations

This study has the advantages of being a large case series of orbital complications of sinusitis encompassing five stages, in which the risk factors, manifestations, diagnosis, treatment, and outcomes were analyzed statistically in conjunction with stage. Most importantly, this study suggests useful ophthalmological indicators for assessing disease staging and prognosis. One limitation of the current study is that we only included hospitalized patients. The exclusion of those with mild preseptal cellulitis that were treated on an outpatient basis unavoidably resulted in an underestimation of the relative frequency of this stage. Another limitation is the retrospective design, which is inherently subject to inaccuracies or inconsistencies in terms of the data obtained and the manner in which they are interpreted or documented.

## Conclusion

Orbital complications of sinusitis tended to occur in children and aged individuals, and diabetes mellitus predisposed patients to more severe disease stages. EOM limitation and proptosis predicted postseptal involvement, and reduced visual acuity and the presence of RAPD indicated the likelihood of intracranial extension which poses a risk of blindness and death. Cooperative consultation between ophthalmologists, otorhinolaryngologists, and neurologists is advisable. Urgent CT or MRI to delineate the extent of disease, intravenous antimicrobial therapy adjusted based on culture results, and prompt surgical drainage when indicated are essential to an optimal prognosis.
